# Multi-omics of cockroaches infected with *Salmonella* Typhimurium identifies molecular signatures of vector colonization

**DOI:** 10.1186/s12864-025-12333-y

**Published:** 2025-11-18

**Authors:** Diing DM Agany, Eduardo A. Callegari, Maria D. Paez, Jose E. Pietri

**Affiliations:** 1 Department of Entomology, Center for Urban and Industrial Pest Management, Purdue University, West Lafayette, IN USA; 2 Division of Biomedical and Translational Sciences, Sanford School of Medicine, University of South Dakota, Vermillion, SD USA; 3 Institute of Inflammation, Immunology and Infectious Disease, Purdue University, West Lafayette, IN USA; 4 Department of Biological Sciences, Purdue University, West Lafayette, IN USA

**Keywords:** Salmonella, Cockroach, Vector, Transcriptomic, Proteomic

## Abstract

**Background:**

German cockroaches (*Blattella germanica*) are prevalent indoor pests that have long been associated with the spread of enteric human pathogens. Recent work investigating the relationship between these insects and *Salmonella enterica* serovar Typhimurium (*S.* Typhimurium), a model pathogen of global concern, demonstrated that *S.* Typhimurium colonizes the cockroach gut. *S*. Typhimurium has a broad host range, but mechanistic molecular insight into how ecologically relevant invertebrate hosts interact with this pathogen is lacking. Here, we applied a multi-omic (transcriptomic and proteomic) approach to infected cockroaches to examine the molecular variables that govern cockroach-borne *S.* Typhimurium transmission.

**Results:**

Our results reveal enriched monocarboxylic acid transport and metabolism, increased long-chain fatty acid transport, increased triglyceride metabolism, and an increased response to reactive oxygen species and free radicals as host signatures of a metabolic shift in the cockroach gut during infection. Surprisingly, downregulation of the immune deficiency (IMD) pathway transcription factor relish, and upregulation of xenobiotic detoxification (glutathione-s-transferase) and known allergens (Blag5 & Blag8, myosin, tropomyosin) were also evident in infected guts.

**Conclusions:**

To our knowledge, this study is both the first omics study of enteric human pathogen infection in a cockroach vector and the first omics study of *S.* Typhimurium in an ecologically relevant insect host, representing a seminal contribution to the field of vector-borne infectious disease. This work provides novel fundamental knowledge regarding the response of insect hosts to *S.* Typhimurium infection and the evolution of vector-pathogen relationships with the potential to inform mitigation of the public health impacts of cockroaches.

**Supplementary Information:**

The online version contains supplementary material available at 10.1186/s12864-025-12333-y.

## Background

Synanthropic cockroaches, including the German cockroach, *Blattella germanica*, have been historically associated with the spread of a variety of enteric human pathogens, such as *Salmonella* spp., *Shigella* spp., and *Vibrio* spp., and are frequently found to harbor these in nature [1–3]. Although cockroaches have traditionally been regarded as passive mechanical (non-propagative) vectors of enteric pathogens, recent studies have implicated German cockroaches as active biological (propagative) vectors of *Salmonella enterica* serovar Typhimurium (*S. *Typhimurium) [4–8], a model bacterial pathogen and global public health concern [9–13]. Collectively, this work has challenged a paradigm in the field of vector-borne infectious disease and spurred renewed interest in investigating the mechanisms of vector-borne transmission of *S. *Typhimurium, which remain poorly delineated and underappreciated despite the global prevalence of both cockroaches and non-typhoidal Salmonellosis.

*S. *Typhimurium has a broad host range, naturally colonizing a variety of mammals, birds, reptiles, and insects [14]. When cockroaches ingest *S. *Typhimurium from infectious material (e.g., feces), the bacteria undergo multiple phases of expansion in the cockroach gut, persisting there for at least seven days and disseminating in cockroach feces without eliciting pathogenesis [5, 6]. The colonization process is marked by complex interactions between cockroach derived gut factors, the gut microbiota, and *S. *Typhimurium virulence factors. For example, type III secretion systems 1 and 2 (*invA*,* spiB*) are required for successful transmission of *S. *Typhimurium in cockroach feces [5]. The importance of these factors for survival in the cockroach host aligns with what little is known about their known roles in colonization of other insects, such as phytophagous leafhoppers, as well as what is known in mammalian hosts [15–20]. Additionally, type 1 fimbriae (*fimA*) are necessary for the formation of *S. *Typhimurium aggregates in the cockroach gut that enhance infection [7]. Meanwhile, relative to common arthropod vectors, cockroaches encode an expanded repertoire of pathogen-associated molecular pattern (PAMP) receptors and antimicrobial peptides (AMPs), which may enable unique responses to diverse microbial challenges encountered in highly septic habitats [21–23]. Low-throughput studies using qRT-PCR have identified several AMPs that are upregulated in the gut of *B. germanica* specifically following ingestion of *S. *Typhimurium, including two attacins and a blattellicin [6]. By modulating the expression of these protective AMPs, the gut microbiota of *B. germanica* provides colonization resistance against *S. *Typhimurium [8].

Although the recent findings summarized above have provided some mechanistic insight and have begun to illuminate the complexity of the relationship between *S. *Typhimurium and cockroach vectors, mechanistic molecular insight into how this pathogen interacts with cockroaches and other ecologically relevant invertebrate hosts is still sparse and fragmented. Thus, there is a necessity to explore the molecular variables that govern cockroach-borne *S. *Typhimurium transmission in an integrative manner. Understanding this biology will provide novel fundamental knowledge regarding the response of insect hosts to *S. *Typhimurium infection and the evolution of vector-pathogen relationships while potentially informing avenues to mitigate the adverse human health impacts of cockroaches.

Transcriptomic and proteomic studies enable the identification of molecular signatures associated with host-microbe interactions in high-throughput, integrative fashion [24], but no studies of this nature have been previously carried out to explore pathogen infection in cockroaches. Critically, simultaneous transcriptomic and proteomic analyses (multi-omics) enable the assessment of dynamic processes across temporal scales and levels of biological organization [25–28]. This nuance is essential to comprehensively understanding the complex functional systems altered by infection, such as signal processing/transduction and immunometabolism [29–31]. Here, we employed an integrative multi-omic (transcriptomic and proteomic) approach to examine the host molecular signatures associated with *S. *Typhimurium infection of the gut of the German cockroach vector. To our knowledge, this is both the first omics study of enteric human pathogen infection in a cockroach vector and the first omics study of *S. *Typhimurium in an ecologically relevant insect host.

## Methods

### Cockroach rearing and Salmonella infections

This study used the American Cyanamid Orlando laboratory strain of German cockroach (*Blattella germanica*). Colonies were reared in plastic containers at 25 ± 1 °C and 40–45% relative humidity on a 12:12 (L: D) hour photoperiod, provided with egg carton harborages, dog chow (Purina, St. Louis, MO, USA), and tap water. From this colony, 50 adult male cockroaches were selected, divided into two equal groups of 25, and placed in separate experimental enclosures. The groups were starved of food and water for three days to subsequently promote uniform oral infection with *S. *Typhimurium (reference strain 14028s) [32]. After the starvation period, cockroaches from one group were fed a stationary phase culture of *S. *Typhimurium at a concentration of OD600 = 1, which results in ingestion of an average of 3.58 × 10^6^ viable bacteria per insect, a naturally achievable dose [5]. These cockroaches were labelled group I (infected). Cockroaches from the second group were fed sterile LB medium only and were labeled group N (non-infected control). Feeding was conducted for 30 min, after which any unfed cockroaches were excluded. Six-hours post feeding, whole guts were dissected and stored individually in TRIzol reagent (ThermoFisher, Waltham, MA) at −80 °C for later extraction of RNA and protein. This timepoint was chosen based on previous work demonstrating bacterial replication and upregulation of several immunity-related genes in orally infected cockroaches by qRT-PCR within this timeframe [6].

### Cockroach transcriptomics: RNA extraction, sequencing, and transcriptome assembly

Total RNAs were isolated from the gut samples of individual cockroaches using TRIzol reagent according to the manufacturer’s protocol. The RNA concentration was then measured using a Qubit fluorometer (ThermoFisher Scientific, Waltham, MA, USA). Ten samples (*N* = 10) from each cockroach group (I and N) underwent sequencing and analysis as follows. 400–1000 ng of total RNA were used for rRNA removal with the Ribo-Zero Plus rRNA Depletion Kit (Illumina, San Diego, CA). Libraries were prepared from rRNA-depleted samples using the KAPA mRNA HyperPrep Kits (Roche, Indianapolis, IN) following the manufacturer’s instructions. After library preparation, the final concentration of the libraries was again measured using a Qubit fluorometer and the average library size was determined using an Agilent 2100 Bioanalyzer (Agilent Technologies, Santa Clara, CA) (Supplementary Table 1). The libraries were then pooled in equimolar ratios of 0.6 nM and sequenced paired-end for 300 cycles on a NovaSeq 6000 system (Illumina).

The quality of the raw reads was assessed using FastQC [33] to identify potential issues in the raw data (e.g., low-quality reads, adapter contamination, or biases) before downstream analysis. Sequences were then trimmed and adapters removed using BBSplite (https://sourceforge.net/projects/bbmap/). Next, cockroach-derived reads were separated (binned) from bacterial reads derived from the abundant microbiome by aligning to the genome of *B. germanica* (GCA_000762945.2) [34]. These raw cockroach reads were then screened for rRNA and any residual rRNA was removed using SortMeRNA [35] against combined databases (16s SILVA v38.1, LSURef, SSURef, and rfam-5.8 S, rfam-5s) [36] accessed on 07/02/2024. The reads were ultimately re-evaluated for quality with FastQC, and the quality metrics were aggregated using the MultiQC tool [37] (Supplementary Fig. 1) (Supplementary Table 1).

**Fig. 1:**
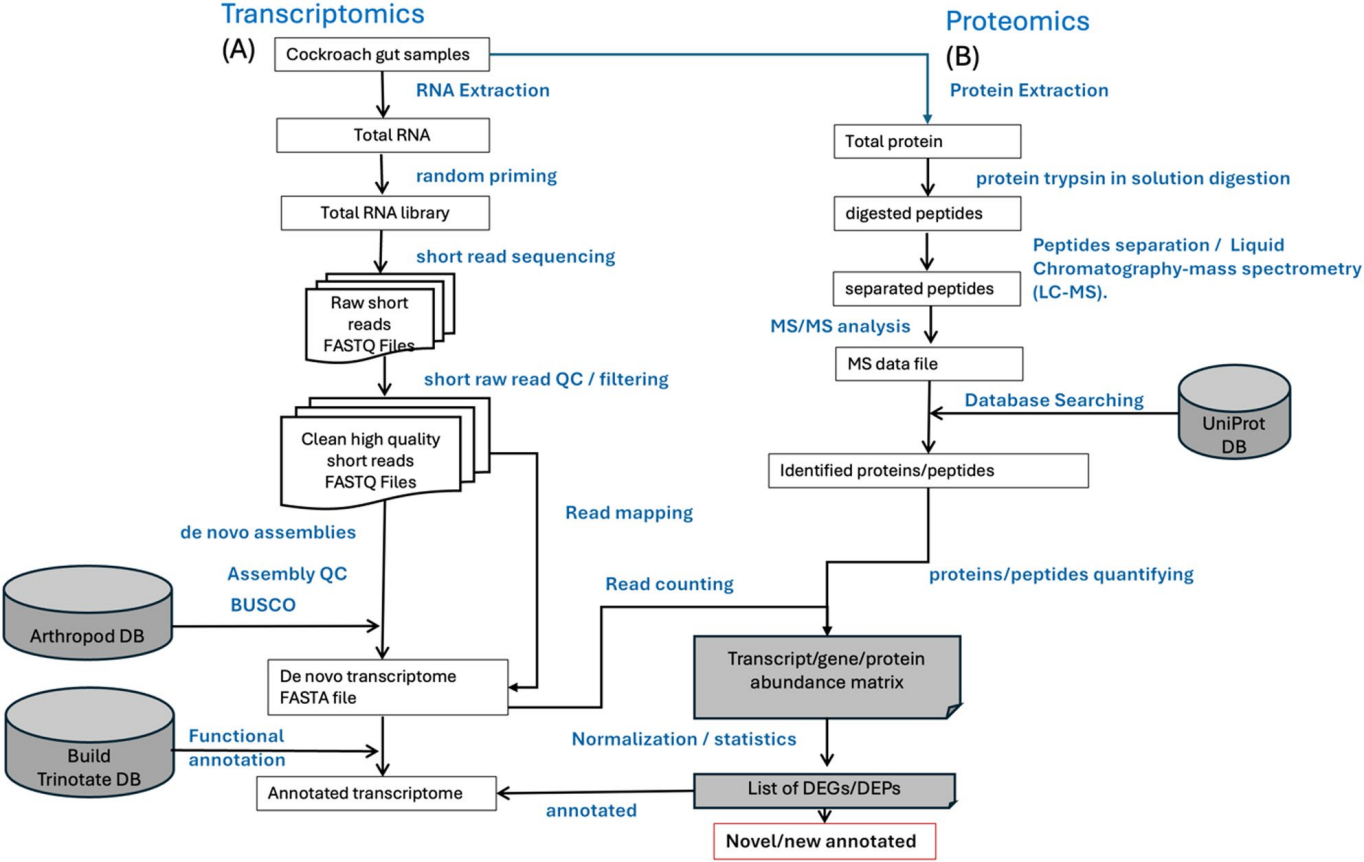
Overview of data analysis

Cleaned cockroach RNA sequences were eventually used for differential expression analysis (DEA) (Fig. 1). *B. germanica* is a non-model organism, and its genome is currently in a low-quality assembly stage (draft/contigs/scaffolds), inadequately annotated, and flagged for contamination, making it unsuitable as a reference for transcriptome assembly. In the absence of an adequate reference genome or transcriptome, *de novo* transcriptome assembly is the preferred approach for DEA [38, 39], and several recent studies of *B. germanica* have followed this approach [22, 23, 40]. Consequently, cleaned cockroach reads were assembled *de novo* using trinity v2.9.1 [41], a widely utilized *de novo* assembly tool for reconstructing full-length transcripts from short RNA-seq reads, particularly for organisms lacking a well-annotated reference genome. In this process, reads were consolidated into extended sequences (contigs) utilizing a de Bruijn graph methodology, subsequently refined into components, and ultimately into isoforms through sequential modules (Inchworm, Chrysalis, and Butterfly) with default parameters, including read normalization and the gcBias parameter activated. This process generated a FASTA file of assembled transcripts, referred to as the “*de novo* transcriptome” or “reference transcriptome,” for subsequent DEA. The quality of the assembly was assessed by the percentage of raw reads that aligned back to the assembled transcriptome (*de novo* transcriptome contigs) using Bowtie2 v2.5 [42] in default mode. The completeness of the assembled transcriptome was evaluated using Benchmarking Universal Single-Copy Orthologs (BUSCO v5.8.0) [43] and the arthropod lineage database (arthropoda_odb10).

### Cockroach transcriptome annotation

Following quality assessment, the *de novo* assembled transcriptome was annotated using Trinotate, a comprehensive annotation suite designed for Trinity-assembled transcripts [44]. Trinotate amalgamates various robust bioinformatics tools and databases to furnish functional insight, including the nature of assembled transcripts, their prospective protein products, and their roles in biological processes, molecular functions, and cellular components. Prerequisite files for constructing the Trinotate database were produced utilizing various annotation tools and methodologies: TransDecoder (http://transdecoder.sf.net) was used to predict coding regions of the transcriptome, producing a FASTA file of amino acid sequences that were predicted to encode proteins. TMHMM v3 [45] and SignalP6 [46] were employed to predict transmembrane domains and signal peptides, respectively. BLASTP (E-value, 1e-5) [47] was used for sequence similarity searches against the SwissProt protein database (downloaded: 05–29-2024). BLASTX [47] was used to search the nucleotide sequences against the non-redundant (nr) protein database. HMMER [48] was used for the identification of protein domains utilizing the Pfam-A.hmm [49] database. All tools/processes were run in default mode unless otherwise stated. These tools (TransDecoder, TMHMM, SignalP6, and HMMER) generate various output files that are consolidated by the Trinotate suite to construct a Trinotate database. The constructed database was utilized for annotating the *de novo* assembled transcriptome (e.g., GO and KEGG identifiers).

### Cockroach proteomics: protein extraction and identification by mass spectrometry

The same cockroach gut samples used in our transcriptomic analysis were also used for proteomic analysis (Fig. 1). Proteins were isolated from these using TRIzol reagent according to the manufacturer’s protocol and the protein pellets were dissolved in 10% SDS. Samples were processed through detergent removal spin columns (Pierce, ThermoFisher) and total protein concentrations were measured with a BCA Protein Assay kit (Pierce, ThermoFisher). Then, in-solution digestion was performed as follows. First, salt concentration and pH were adjusted to the requirements for Trypsin protease (Promega, Madison, WI) using 100 mM ammonium bicarbonate/5% acetonitrile at pH = 8. Then, reduction and alkylation were performed using 1/10 v/v 50 mM DTT (Sigma-Aldrich, Saint Louis, MO) at 65 °C for 5 min, followed by 1/10 v/v 100 mM Iodoacetamide (Sigma-Aldrich) at room temperature under dark for 30 min. Sequencing grade trypsin (Promega) was used at a concentration of 1:20 (1 ug of trypsin:20 ug of total protein) for in solution digestion at 37 °C overnight and the reaction was stopped by changing the pH to 4 using LC/MS grade Trifluoroacetic Acid (Fisher Chemical, Pittsburgh, PA) at a concentration of 0.5%. Finally, the digested peptides were concentrated on a SpeedVac centrifuge concentrator [50, 51].

Digested peptides were in-line desalted using an Easy nLC 1200 nanoUHPLC (ThermoFisher) through a trapping and desalting online trap column (300 μm x 20 mm Acclaim PepMap C18 100Å (ThermoScientific) and separated by an Easy-Spray PepMap RSLC 2 μm, 75 μm x 15 cm, nanoViper column (ThermoScientific) coupled to nanoESI orbitrap Exploris 240 h/MA mass spectrometer (ThermoFisher). The chromatography condition parameters were set to 0–1 min, 1% B isocratic; 2–60 min, 1–25%; 61–90 min, 25–50%; and 92–101 min, 50–100% B linear mobile phase A (water/formic acid, 99.9:0.1% v/v) and Phase B (water/acetonitrile/formic acid, 20/80/0.1% v/v). The solvent flow rate was 300 nL per min. The Orbitrap Exploris 240 instrument was operated under data-dependent acquisition mode (DDA) in the top 12 modes to switch automatically between full-scan MS and MS/MS acquisition. The collision energy type selected was the normalized option, the HCD collision energy (%) was 30, and the data type was centroid. The ions were analyzed in positive MS ion mode (m/z 375 − 1500) with 120,000 resolution (m/z 200) after accumulation with target ions to 1 × 10^6 value based on predictive AGC. The MS/MS ion selection was set at m/z 65–1800 with 30,000 resolution to 1 × 10^5 counts [52].

The spectra were deconvoluted and analyzed using Mascot Distiller v2.6 (www.matrixscience.com) and Proteome Discoverer v2.5 (ThermoFisher). A mascot generic format list (MGF format) was generated to identify + 1 or multiple charged precursor ions from the MS data file. MASCOT server v2.8.1 (www.matrix-science.com) in MS/MS ion search mode (local license) was applied to conduct peptide matches (peptide masses and sequence tags) and protein searches against the Uniprot *Blattella germanica* database (proteome ID UP000245037; 29,472 sequences; 9,127,135 residues). Searches were conducted using the following parameters: carbamidomethyl (C) on cysteine was fixed, and variable modifications included asparagine and glutamine deamidation, and methionine oxidation. Two missed cleavages were allowed; monoisotopic masses were counted; the precursor peptide and fragment mass tolerance were set at 15 ppm and 0.02 Da, respectively; the ion score, or expected cut-off, was set at 5. The MS/MS spectra were searched with MASCOT (www.matrixscience.com, local license) using a 95% confidence interval (% C.I.) threshold (*p* < 0.05), with which a minimum score of 27 was used for peptide identification, indicating identity or extensive homology, and a FDR < 5% was considered. Redundant proteins were not considered to mitigate potential database contaminants and improve accuracy. Quantitative analysis (spectral counting) was performed using ProteoIQ v2.8 (local license) (www.premierbiosoft.com). Additional details on this method can be found in (Supplementary File 1).

### Transcriptome and proteome quantification and differential expression analysis

Transcript expression was quantified using Salmon-v1.10.3 [53], a quasi-mapper, with default parameters, employing cleaned reads and the *de novo* transcriptome as the reference. This process generated quantified transcript count files (quant.sf files) for each sample (*N* = 10 per group), and it was repeated for gene-level counts, as Salmon can produce both transcript-level (transcripts with isoforms) and gene-level (transcript isoforms collapsed to gene) quantifications. The individual quant.sf files were aggregated to produce expression count matrices for transcript and gene counts. The raw count matrix of gene expression (Supplementary Files 2 & 3) was used for the analysis of differentially expressed genes (DEGs).

On the other hand, the raw count matrix of protein abundance (Supplementary Files 4 & 5) derived from the proteomic methodology outlined in the preceding section was utilized for differential expression protein (DEPs) analyses. Differential gene and protein expression analyses were performed on the two matrices separately in RStudio v2023.12.0.369, Rv4.3.2. using DESeq2 v1.46.0 [54] with default parameter settings. DESeq2 employed a negative binomial generalized linear model to identify genes or proteins that are significantly up- or down-regulated between different experimental conditions (infected vs. non-infected samples). Moreover, it used a median of the ratios to normalize data. Statistical significance thresholds for differentially expressed genes (DEGs) and proteins (DEPs) were established at an adjusted p-value of < 0.05 and an absolute log2 fold change of ≥ 0.65 or 1 for genes and proteins, respectively. Different thresholds within a conventional range were used to reflect the inherent biological differences between transcriptomic and proteomic data. Once significantly differentially expressed genes were identified, InterPro [55], eggNOG-mapper [56], Blast2Go [57], and the STRING database [58] were used to perform functional analysis on the up and downregulated cockroach DEGs in addition to the previous general Trinotate annotation. The input for these tools is a FASTA file containing significantly differentially expressed gene sequences, identified by Trinity IDs, such as TRINITY_DN39262_c2_g1. UniProt protein identifiers for significant DEPs were obtained, and ShinyGO [59] was utilized to perform GO functional analysis on the DEGs and DEPs. Reduce Visualize Gene Ontology (REVIGO) was used to remove redundant and obsolete GO terms.

## Results

### Differential gene and protein expression between uninfected cockroaches and cockroaches infected with *S*. Typhimurium

To investigate the effects of *S. *Typhimurium on cockroaches and their molecular responses to infection, we first employed bulk RNA sequencing (transcriptomics) and mass spectrometry (proteomics) to compare global changes in RNA and protein levels in gut samples from infected and uninfected cockroaches. Initially, the transcriptomic and proteomic datasets were evaluated independently. An average of 11,864,290 cockroach-derived RNA reads per sample were obtained after data cleaning (Supplementary Table 1). The reads were *de novo* assembled into a reference transcriptome, achieving a 97.6%. BUSCO completeness. At the protein level, an average of 10.93% of the 30, 094 protein proteome was detected in uninfected gut samples, and an average of 11.3% was detected in infected gut samples.

**Fig. 2:**
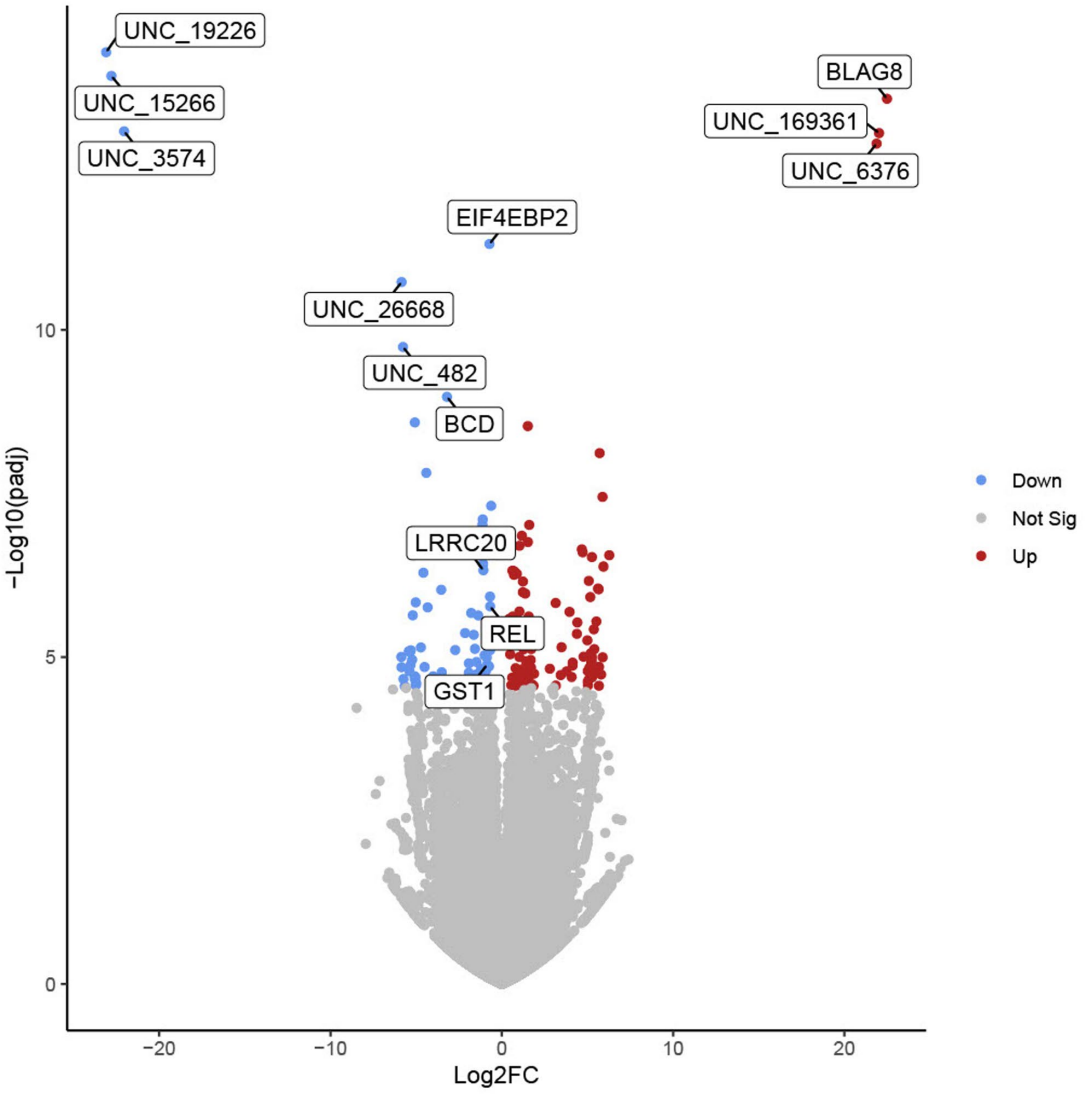
Differentially expressed genes (DEGs) in cockroaches infected with *S. *Typhimurium relative to uninfected cockroaches. Downregulated genes are shown in blue, upregulated in red, and non-significant in gray based on Benjamini-Hochberg (B&H) adjusted p-value and log2 fold change cutoffs of < 0.05 and of ≥ 0.65, respectively. The 10 most significant DEGs and genes of interest are labeled with gene IDs. *N*=10 cockroaches per group (infected and non-infected)

**Fig. 3:**
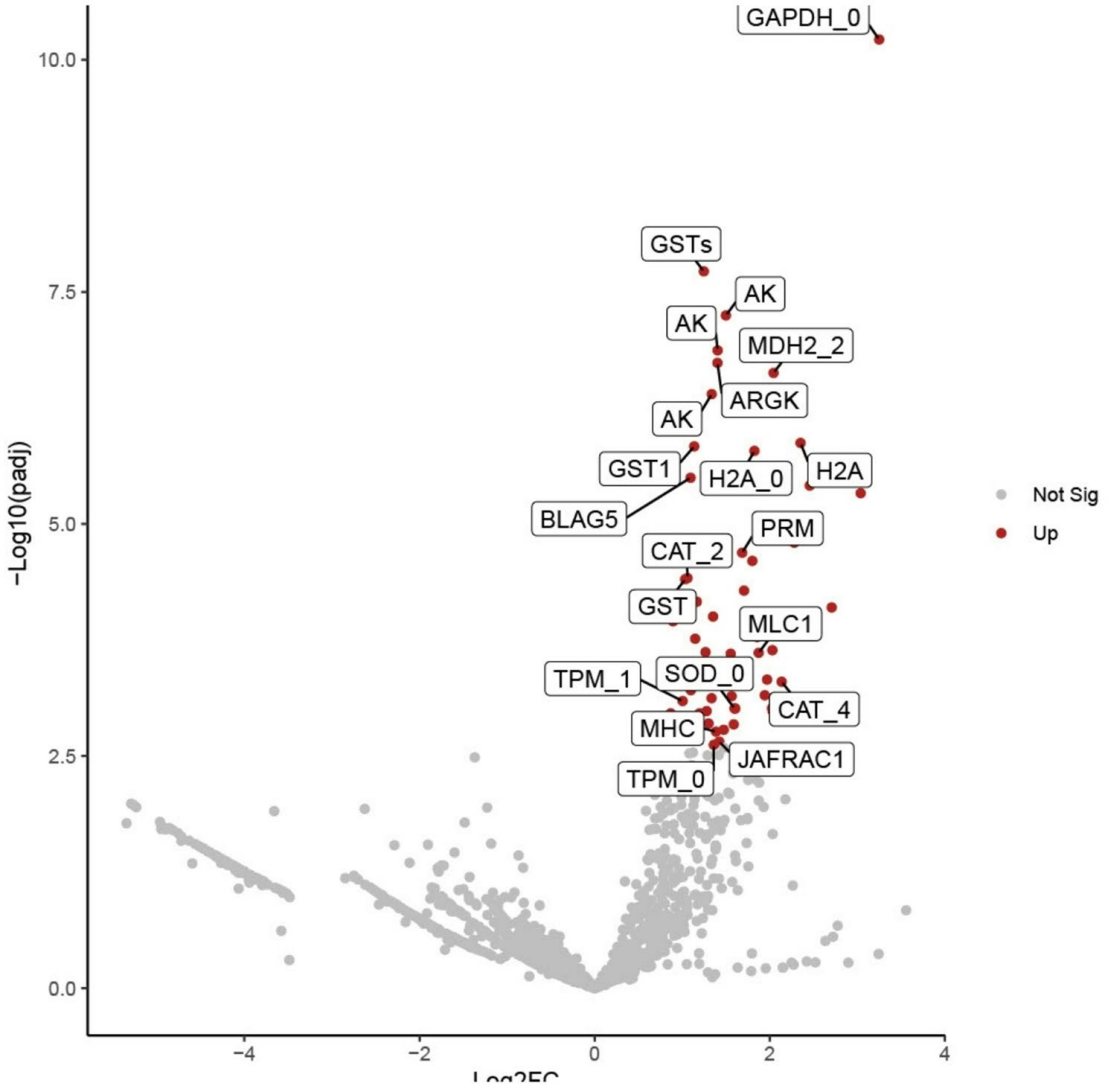
Differentially expressed proteins (DEPs) in cockroaches infected with *S.*Typhimurium relative to uninfected cockroaches. Significantly upregulated proteins are shown in red and non-significant in gray based on Benjamini-Hochberg (B&H) adjusted p-value and log2 fold change cutoffs of <0.05 and of ≥1, respectively. The 10 most significant DEPs and proteins of interest are labeled with their protein ID, and UniProt protein IDs are included in table 2 and supplementary table 6. No significantly downregulated proteins were identified to display. N=8-10 cockroaches per group (infected and non-infected)

Differentially expressed genes (DEGs) and differentially expressed proteins (DEPs) were identified utilizing DESeq2, applying a log2 fold change threshold of ≥ 0.65 for genes and ≥ 1 for proteins, and an adjusted P value of < 0.05 for both. This resulted in the identification of 105 significantly upregulated 79 significantly downregulated cockroach genes in infected individuals when compared to uninfected controls (Fig. 2). A full list of these DEGs, their values, and sequences are provided in (Supplementary Tables 2 & Supplementary File 6). For protein expression, 62 cockroach proteins were upregulated in infected individuals relative to controls, and no downregulated proteins were identified (Fig. 3, Supplementary Table 3).

### Functional annotation of differentially expressed cockroach genes

**Fig. 4:**
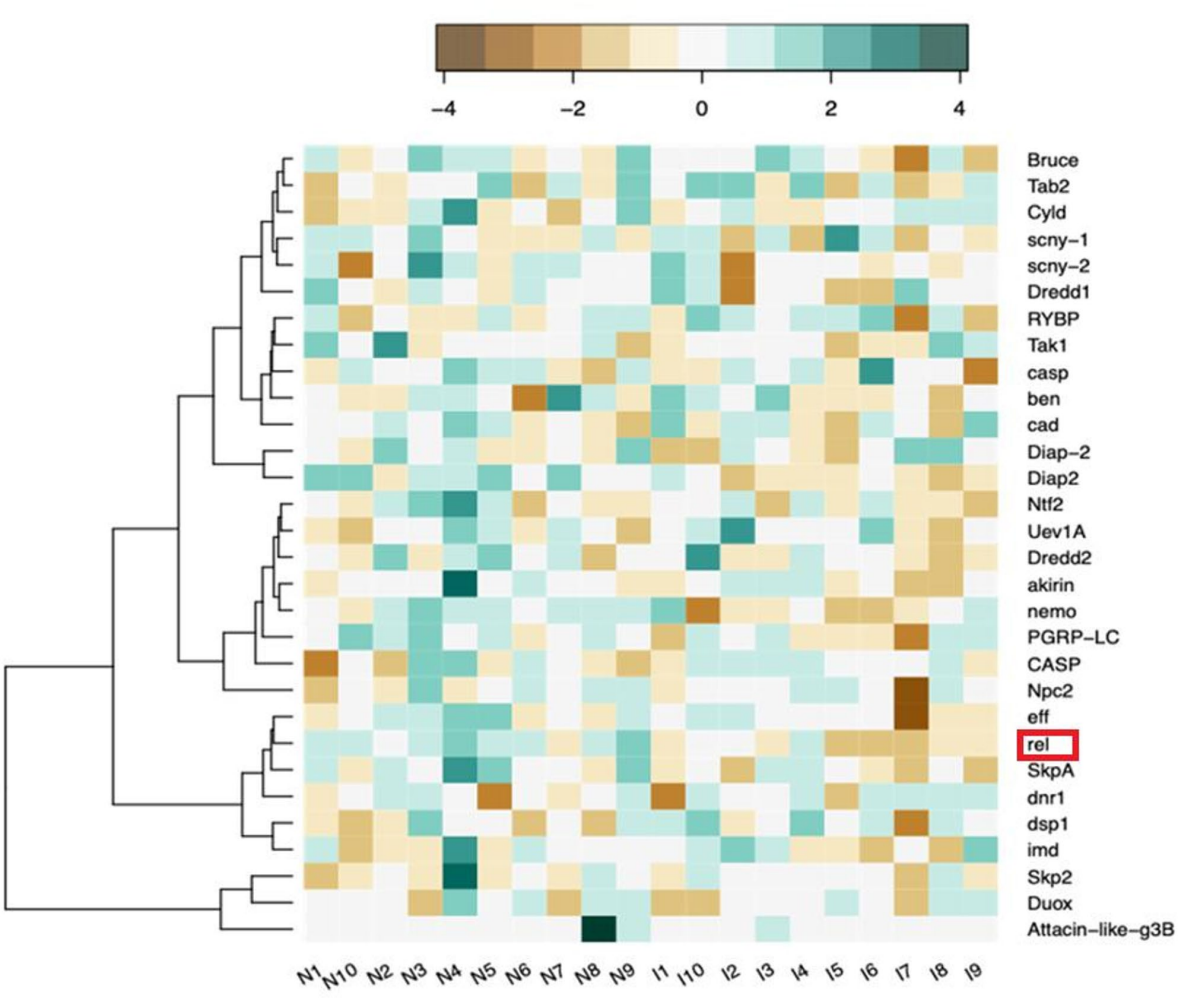
Heatmap of expression of IMD pathway genes in infected and non-infected cockroach transcriptomes. The color key indicates log-scaled expression values. *N*= 10 cockroaches per group. Infected cockroaches are labelled “I” and non-infected cockroaches are labelled“N.” Note *relish* gene expression (rel), red box 8^th^ from the bottom

To begin to biologically understand the response of cockroaches to *S. *Typhimurium infection, we examined the functions of the differentially expressed genes and proteins that we identified (Tables 1 and 2). Various methods allowed for annotation of 35 DEGs to protein family, KEGG pathway, cluster of orthologous groups (COG), or gene ontology (GO) categories (Table 1, Supplementary Table 2). Of these 35 annotated DEGs, 20 are significantly upregulated genes and 15 are significantly downregulated genes. The most significantly upregulated DEGs were *blag8* (22.5 log2 FC), *UNC_169361* (22.0 log2 FC), and *UNC_6376* (21.9 log2 FC). *Blag8* is annotated as an allergen gene, consistent with the upregulation of proteins BLAG5 and BLAG8 in infected cockroaches at the proteomic level (Tables 2 and 3). The two others are signaling proteins with specific functions uncharacterized. On the other hand, the most significantly downregulated DEGs were *UNC_19226* (−23.1, log2 FC), *UNC_15266* (−22.8 log2 FC), and *UNC_3574* (−22.0 log2 FC). We annotated these genes as signaling or transmembrane proteins, but their specific functions also remain uncharacterized. Interestingly, *relish,* a transcription factor gene canonically associated with regulation of the immune deficiency (IMD) pathway mediated immune response, was significantly downregulated in infected cockroaches. We also identified expression of 30 IMD pathway genes [22, 23] in our data, although these genes were not differentially expressed at the < 0.05 adjusted p-value cutoff, with the exception of the *relish* gene (Fig. 4, Supplementary Tables 4 & 5). Furthermore, *gstd1*, a glutathione-s-transferase, was significantly downregulated, whereas GSTσ and GST1 levels appeared significantly upregulated in the proteomic data (Tables 2 and 3).

**Table 1:** Selected annotated cockroach genes differentially expressed in *S.* Typhimurium infection. Additional information on all differentially expressed genes is available in Supplementary Table 2

Trinity Gene ID	Log2FC	Padj	Annotated Gene	pfam
TRINITY_DN39262_c2_g1	22.483	3.27E-09	*blag8*	Ins_allergen_rp
TRINITY_DN32215_c1_g1	1.8773	0.0402	*slc20a2*	PHO4
TRINITY_DN4020_c1_g1	1.3566	0.0244	*fusl*	Fuseless
TRINITY_DN32841_c2_g1	1.3298	0.039	*zbed5*	zf-BED
TRINITY_DN13750_c0_g1	1.0436	0.048	*gnb2l1*	WD40
TRINITY_DN2652_c2_g2	1.0240	0.0106	*plekhg4*	PH, RhoGEF
TRINITY_DN2062_c1_g1	−0.683	0.0095	*rel*	Relish
TRINITY_DN4591_c10_g1	−0.721	2.34E-07	*eif4ebp2*	eIF_4EBP
TRINITY_DN34_c4_g2	−0.817	0.0362	*cib*	Thymosin
TRINITY_DN2078_c0_g1	−0.861	0.0354	*gstd1*	GST_C, GST_C_2,GST_C_3,GST_N, GST_N_3
TRINITY_DN14769_c0_g1	−1.075	0.0041	*lrrc20*	LRR_1,LRR_8
TRINITY_DN345_c0_g1	−1.135	0.0014	*picot*	MFS_1
TRINITY_DN34159_c0_g1	−1.569	0.0263	*polr3e*	Sin_N
TRINITY_DN100940_c0_g1	−3.200	0.000035	*bcd*	Acyl-CoA_dh_1,Acyl-CoA_dh_M, Acyl-CoA_dh_N
TRINITY_DN25783_c1_g2	−3.944	0.0443	*pogk*	BrkDBD, DDE_1,HTH_Tnp_Tc5,KRAB

**Table 2:** Selected annotated cockroach proteins differentially expressed in *S. *Typhimurium infection. Additional information on all differentially expressed proteins is available in Supplementary Table 3

UniProt ID	Log2FC	Padj	Name	Protein ID
A0A2P8ZEJ5	3.2482471	7.89E-08	Glyceraldehyde-3-phosphate dehydrogenase	GAPDH_0
A0A2P8YBA0	2.13502145	0.01637937	cat_4 - Catalase	CAT_4
A0A2P8XGS3	2.04233931	5.1385E-05	Malate dehydrogenase	MDH2_2
A0A2P8ZP67	1.8724323	0.01021533	Myosin light chain alkali	MLC1
A0A2P8XJ80	1.8240042	0.00021281	Histone H2A	H2A_0
A0A2P8ZN71	1.68431104	0.00147921	Paramyosin	PRM
A0A2P8XTT4	1.605828199	0.024925778	Calcium-transporting ATPase	Ca-P60A
A0A2P8ZBC0	1.59609852	0.02492578	Superoxide dismutase [Cu-Zn]	SOD_0
B9VAT1	1.49857845	2.47E-05	Arginine kinase	AK
A0A2P8Z9A3	1.42202086	0.04515907	Peroxiredoxin 1	JAFRAC1
G9JWG6	1.41565422	0.01441358	Allergen bla g 8	BLA G 8
Q2HZF2	1.40376097	4.40E-05	Arginine kinase	AK
A0A2P8XGR1	1.40194661	4.82E-05	Arginine kinase ARGK	ARGK
A0A2P8XQ42	1.38534321	0.0369707	Myosin heavy chain	MHC
A0A2P8YRT0	1.36008556	0.04817588	Tropomyosin	TPM_0
G8XWV9	1.33594713	7.4534E-05	Arginine kinase	AK
G8XWV7	1.24597592	1.24E-05	Glutathione S transferase class sigma	GSTs
A0A2P8ZEL2	1.19773731	0.02606536	Glyceraldehyde-3-phosphate dehydrogenase	GAPDH_1
A0A2P8ZI29	1.13638915	0.00021168	Glutathione S-transferase GST1	GST1
A9XFW9	1.09484692	0.00034684	Bla g 5 variant allergen	BLAG 5
A0A2P8Z956	1.05951347	0.00245393	Catalase cat_2	CAT_2
A0A2P8YR56	1.025617853	0.001225382	Apolipophorin	APLP
O18598	1.03306277	0.00245393	Glutathione S-transferase	GST
A0A2P8XCT5	1.00433097	0.02296178	Tropomyosin TPM_1	TPM_1

**Table 3:** Candidates differentially regulated in both the transcriptome and proteome. Differentially expressed genes were translated and the products mapped to differentially expressed proteins

Trinity Gene ID/UniProt ID	Gene ID/Protein ID	Regulated Down/Up
*TRINITY_DN2078_c0_g1/*A0A2P8ZI29	*gstd1/*GST1	Down/Up
*TRINITY_DN2078_c0_g1/*G8XWV7	*gstd1/*GST-s	Down/Up
*TRINITY_DN2078_c0_g1/*O18598	*gstd1/*GST	Down/Up
*TRINITY_DN39262_c2_g1/*G9JWG6	*blag8/*BLAG8	Up/Up
*TRINITY_DN4020_c1_g1/*A0A2P8XTT4	*fusl/*Ca-P60A	Up/Up

When BLASTx was used to search the remaining 149 unannotated DEGs across various databases, additional differentially expressed genes (DEGs) were assigned putative functions. These included the *UNC_181674*, which matched hypothetical proteins C0J52_08276 from *B. germanica*, *UNC_888*, *UNC_14836*, and *UNC_20833*, which matched hypothetical protein DMN91_004009 from *Ooceraea biroi*, hypothetical protein AVEN_106333-1 from *Araneus ventricosus*, and an unnamed protein product from *Peronospora matthiolae*, respectively. Also, *UNC_14836* is annotated to contain domain DUF4817 (HTH_32) with a DNA-binding function. Moreover, BLASTN mapping to the *B. germanica* genome (GCA_000762945.2; Bger_2.0) revealed that several differentially expressed genes (DEGs) in our data originated from unplaced contigs of the genome. These sequences are presently not annotated to any known functions, making them possibly novel genes/proteins that might be involved in infection and immunity processes in cockroaches.

### GO enrichment analysis of differentially expressed cockroach genes

**Fig. 5:**
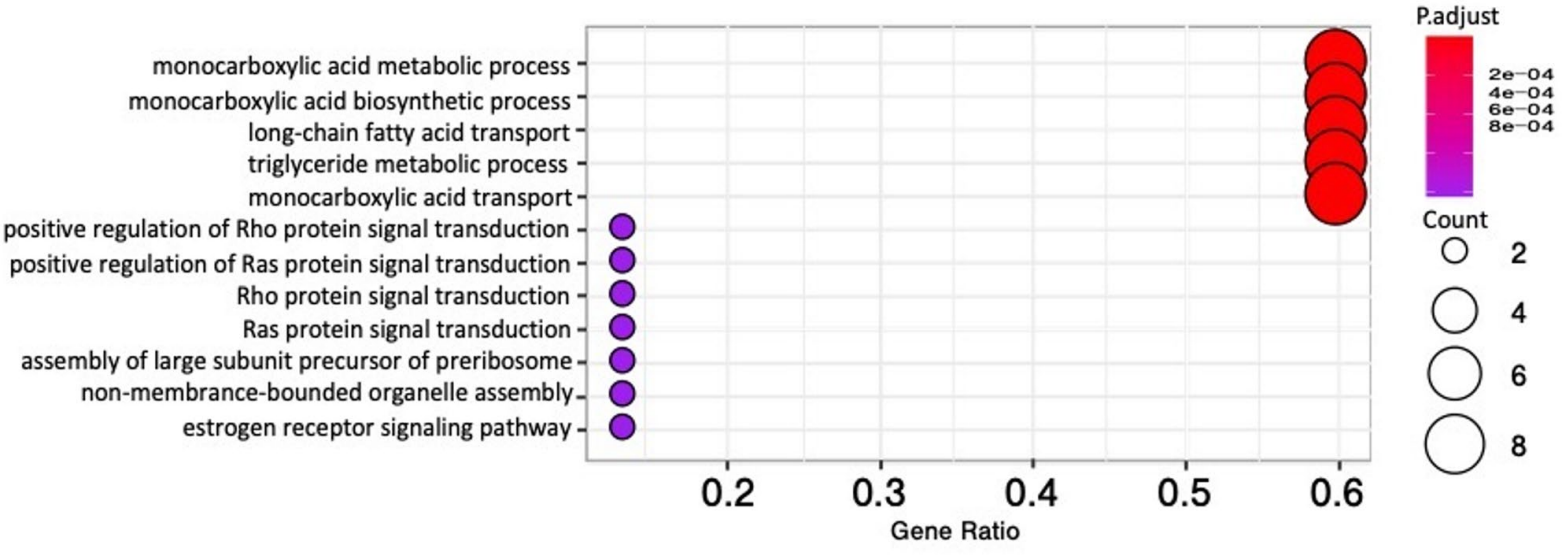
GO enrichment analysis of cockroach DEGs upregulated during *S. *Typhimurium infection. Shown are the top biological processes enriched among 105 significantly upregulated cockroach genes as determined by the hypergeometric test and enrichment FDR < 0.05. Dot size represents the number of genes enriched within each term while statistical significance of enrichment is indicated by dot color. The X-axis denotes the proportion of genes within the GO category that were significantly upregulated. Additional details are provided in Supplementary Table 5

The Blast2Go program was employed to conduct GO enrichment analysis of the significant DEGs to further integratively understand the molecular signatures of *S. *Typhimurium infection of the cockroach gut. While the downregulated DEG set did not yield any enrichment, among the 105 significantly upregulated DEGs, the most significantly enriched GO terms in infected cockroaches pertain to the biological processes of monocarboxylic acid metabolism, monocarboxylic acid biosynthesis, monocarboxylic acid transport, long-chain fatty acid transport, and triglyceride metabolism (Fig. 5, Supplementary Table 6).

### GO enrichment analysis of differentially expressed cockroach proteins

**Fig. 6:**
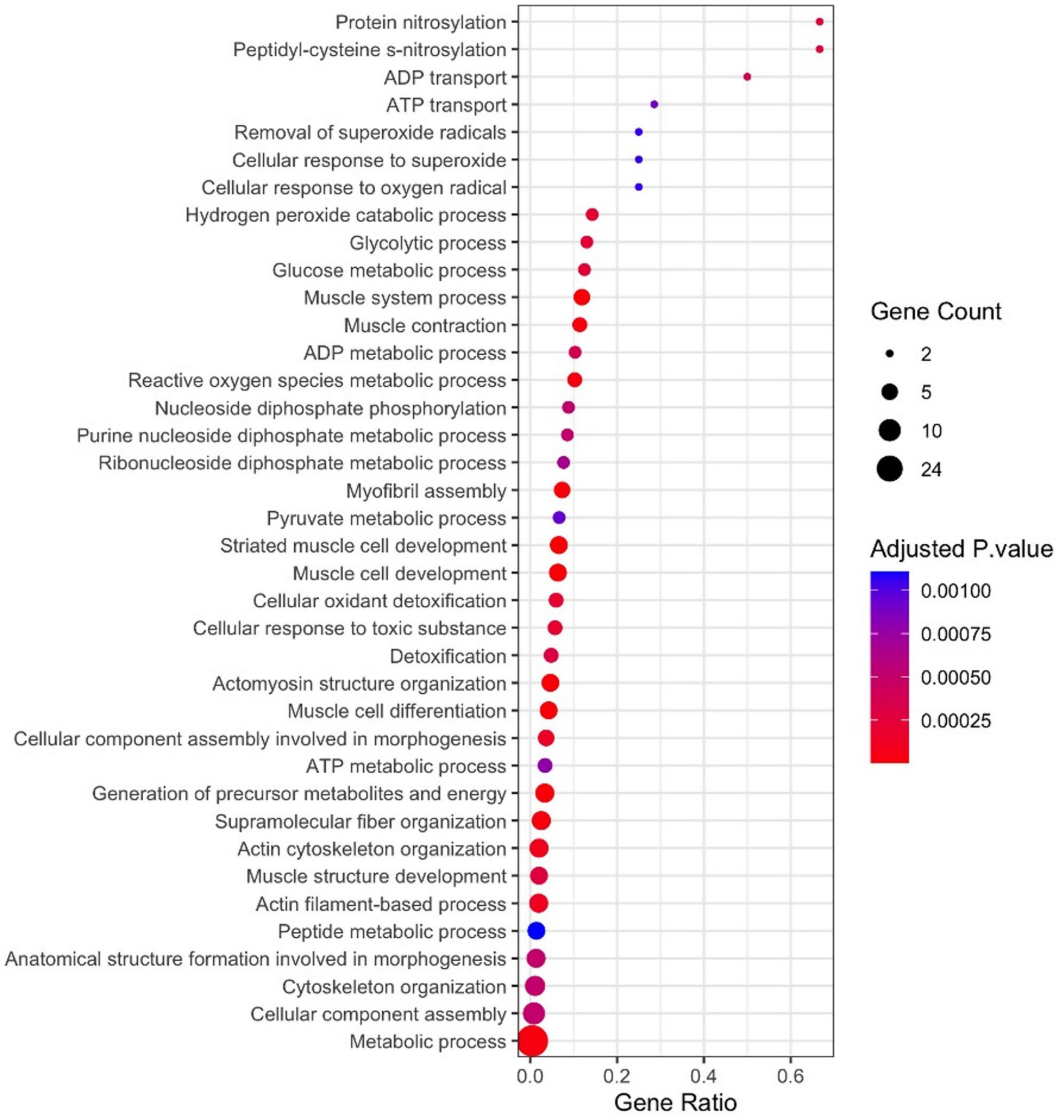
GO enrichment analysis of cockroach proteins upregulated during *S. *Typhimurium infection. Shown are the top 38 biological processes enriched among 62 significantly upregulated cockroach proteins as determined by the hypergeometric test and enrichment FDR < 0.05. Dot size represents the number of genes enriched within each term while statistical significance of enrichment is indicated by dot color. The X-axis denotes the proportion of genes within the GO category that were significantly upregulated. Additional details are provided in Supplementary Table 6

We next conducted a GO enrichment analysis on the 62 proteins that were significantly upregulated in *S. *Typhimurium infected cockroaches relative to uninfected controls. S-nitrosylation, peptidyl-cysteine s-nitrosylation, and ADP transport are the top three biological processes found to be enriched in the 62 protein set (Fig. 6, Supplementary Table 7). Processes related to the response and removal of reactive oxygen species and free radicals (e.g., hydrogen peroxide catabolism, removal of superoxide) were also found to be enriched, including the JAFRAC1 (peroxiredoxin), SOD_0 (superoxide dismutase), CAT_4, and CAT_2 (catalase) proteins. Furthermore, the enrichment of several muscle related processes appears to reflect the upregulation of myosin and tropomyosin proteins, which are known human allergens [60]. BLAG8 is known to be a myosin chain.

## Discussion

The results presented here provide novel molecular insight into the responses of an ecologically relevant insect host to *S. *Typhimurium infection. These findings highlight biological processes and pathways that may ultimately drive the success of colonization and downstream vector-borne transmission via fecal shedding, identifying specific targets for further investigation. Several phenomena with important implications are evident from our data.

Regarding the cockroach response to infection, it is interesting to note that there was no strong signal of IMD-mediated antibacterial immunity, which is typically activated upon ingestion of Gram-negative peptidoglycan in other insects such as fruit flies and silkworms [61, 62]. Instead, expression of the transcription factor *relish* gene, which controls IMD-mediated production of effectors such as AMPs, was significantly downregulated. As *relish* is the most downstream component of the IMD pathway and acts as a master transcriptional regulator, this strongly indicates that *S. *Typhimurium can inhibit innate immunity in the insect gut as it does in mammals. In our previous studies, we have observed upregulated expression of some AMPs, such as *attacin 2*, in the cockroach gut within the first hour of ingestion of *S. *Typhimurium [6]. Further, RNAi knockdown of *attacin 2* expression revealed that this AMP antagonizes *S. *Typhimurium [8]. Together, these findings suggest that suppression of IMD-mediated immunity is an important colonization strategy for *S. *Typhimurium in the cockroach gut. However, our analysis failed to detect expression of many of the known cockroach AMPs in either controls or infected insects [22, 23]. This could be a technical limitation due to the short length of these genes, or a result of time, tissue, sex, or developmental stage-specific expression of the undetected AMPs. Lastly, an apparent homolog of the leucine-rich repeat protein gene *lrrc20* was significantly downregulated in our transcriptome data. Although the exact molecular functions of this gene in other organisms are not completely understood, it is expressed in a variety of tissues including immune cells, and numerous leucine-rich repeat proteins are known to be involved in immunity.

Despite a lack of IMD-mediated immune response, GO enrichment analysis strongly indicated a metabolic shift in the gut of infected cockroaches. This shift manifested in increased monocarboxylic acid transport and metabolism, increased mobilization of long-chain fatty acids, and increased triglyceride metabolism. Metabolic shifts are a hallmark of infection in many organisms. Monocarboxylic acid transport and metabolism are crucial for glycolysis and the TCA cycle, but also for lipid metabolism via beta-oxidation. In fruit flies, bacterial infection reduces triglyceride stores and mobilizes fatty acids [63]. Although this can lead to pathological wasting, it is also important for fueling immune responses via beta-oxidation [63]. Considered alongside the lack of an IMD-mediated immune response, the metabolic shift we observed in cockroaches is likely part of a tolerance response to this infection, as *S. *Typhimurium is not overtly pathogenic to cockroaches even when high doses are ingested and persist [6]. At the proteome level, the enrichment of proteins involved in the breakdown of reactive oxygen species (ROS) and response to free radicals may be a secondary response to the metabolic shifts that take place during infection. For example, beta-oxidation is known to increase the production of ROS [64, 65]. While ROS production itself may also be an immune response that limits *S. *Typhimurium, it must be balanced to prevent cellular damage. For example, Peroxiredoxin (JAFRAC1), upregulated in our proteomic data, maintains redox homeostasis and promotes survival during enteric bacterial infection in fruit flies [66].

The dynamics of GSTs, which are involved in the detoxification of xenobiotics including many insecticides, are of further interest. Cockroaches encode multiple GSTs. In our data, expression of one gene was downregulated in the transcriptome, but multiple GSTs appeared upregulated at the proteome level, reflecting likely differential regulation of distinct GSTs during infection. In a recent transcriptomic study of German cockroaches, selection for resistance to the common insecticide indoxacarb by repeated exposure over six generations reduced the relative abundance of microbial transcripts [40]. The authors of that study hypothesized of regulatory overlap between insecticide resistance and antimicrobial mechanisms. Our data agree with and provide the first direct evidence of this concept by demonstrating that ingestion of bacteria results in changes in GSTs. Whether GSTs have a direct antimicrobial role or are regulated secondarily as a result of the immune response (e.g., as antioxidants) remains to be determined. Nevertheless, this relationship has important implications when considering targeting microbes for cockroach control, as well as the effect that insecticide exposure may have on pathogen transmission. Targeting commensal bacteria may be a useful approach to boost the effectiveness of insecticide treatments by suppressing microbe-linked GSTs [67, 68]. On the other hand, if upregulation of GST is beneficial for bacterial growth, insecticide exposures that upregulate GST may enhance susceptibility to infection by some human pathogens such as *S. *Typhimurium, increasing cockroach vector competence.

The upregulation of two known cockroach-derived human allergens, at both the gene and protein levels in infected guts, strengthens a previously predicted link between microbial infection and allergen production. These results are consistent with two intriguing recent studies showing that antibiotic treatment of cockroaches reduces levels of *blag1*, *blag2*, and *blag5* allergens [69, 70]. Cockroach allergens are shed in the exuviae and feces and contribute to asthma development and morbidity, particularly in children living in infested homes [71]. Multiple lines of evidence now indicate that these are closely connected to the dynamics of microbial populations in the gut. Understanding the mechanisms behind this connection may provide opportunities to mitigate both allergen shedding and pathogen transmission by cockroaches. Thus, this finding should spur renewed interest in investigating the functions of these proteins beyond those that are currently known.

Though entirely novel, our study has several limitations that should be acknowledged. First, to increase sample size, we focused only on a single time point relatively early in infection. Although this time point was specifically chosen and has known biological relevance [5], temporal resolution in our study is limited. It is likely that additional or different responses emerge later in infection, which could be investigated by replicating our study at additional time points. Contributing to the potential for missing some responses, annotation of the genome of *B. germanica* still needs improvement, which complicates analyses of differentially expressed genes and pathways at deeper functional levels. Moreover, the specificity of some of the responses we documented from cockroaches is unknown. Some of these responses may be generalized responses to bacterial infection. Nevertheless, given the dearth of information available on the molecular interactions underlying the relationships between *Salmonella* and ecologically relevant insect hosts such as cockroaches, this information represents a major advance. The insight obtained here will inform controlled mechanistic studies to elucidate the roles of specific pathways in regulating vector-borne transmission of *S. *Typhimurium. In the future, experiments in which cockroach genes of interest are knocked down will be carried out to directly examine their mechanistic roles in infection and transmission dynamics [5, 8].

## Supplementary Information

Supplementary Material 1. Transcriptome and proteome quality metrics and PCA.

Supplementary Material 2. RNA sample metadata.

Supplementary Material 3. List of all cockroach genes differentially expressed in *S. *Typhimurium infected individuals.

Supplementary Material 4. List of all cockroach proteins differentially expressed in *S. *Typhimurium infected individuals.

Supplementary Material 5. IMD pathway expression matrix.

Supplementary Material 6. IMD pathway differential expression analysis.

Supplementary Material 7. GO enrichment of cockroach genes upregulated during infection.

Supplementary Material 8. GO enrichment of cockroach proteins upregulated during infection.

Supplementary Material 9. Additional proteomics methods.

Supplementary Material 10. Cockroach RNASeq raw Trinity counts matrix.

Supplementary Material 11. Cockroach RNASeq normalized Trinity counts matrix.

Supplementary Material 12. Cockroach raw proteome FDR 1 perc results matrix.

Supplementary Material 13. Cockroach proteome normalized FDR 1 perc results matrix.

Supplementary Material 14. Significant differentially expressed genes Nucleotides FASTA File.

## Data Availability

Raw RNA sequencing data were deposited into the NCBI SRA: BioProject PRJNA1223474.Raw proteomic data were deposited into the MassIVE repository (https://massive.ucsd.edu): MSV000097934.
