# Supplementary material for: Multi-omics of cockroaches infected with Salmonella Typhimurium identifies molecular signatures of vector colonization
Source: BMC Genomics. 2025 Nov 18;26:1118. doi: 10.1186/s12864-025-12333-y (PMC12752085; doi:10.1186/s12864-025-12333-y)
Supplement: Supplementary file 1 — Supplementary Material 1. Transcriptome and proteome quality metrics and PCA. [file 12864_2025_12333_MOESM1_ESM.docx]

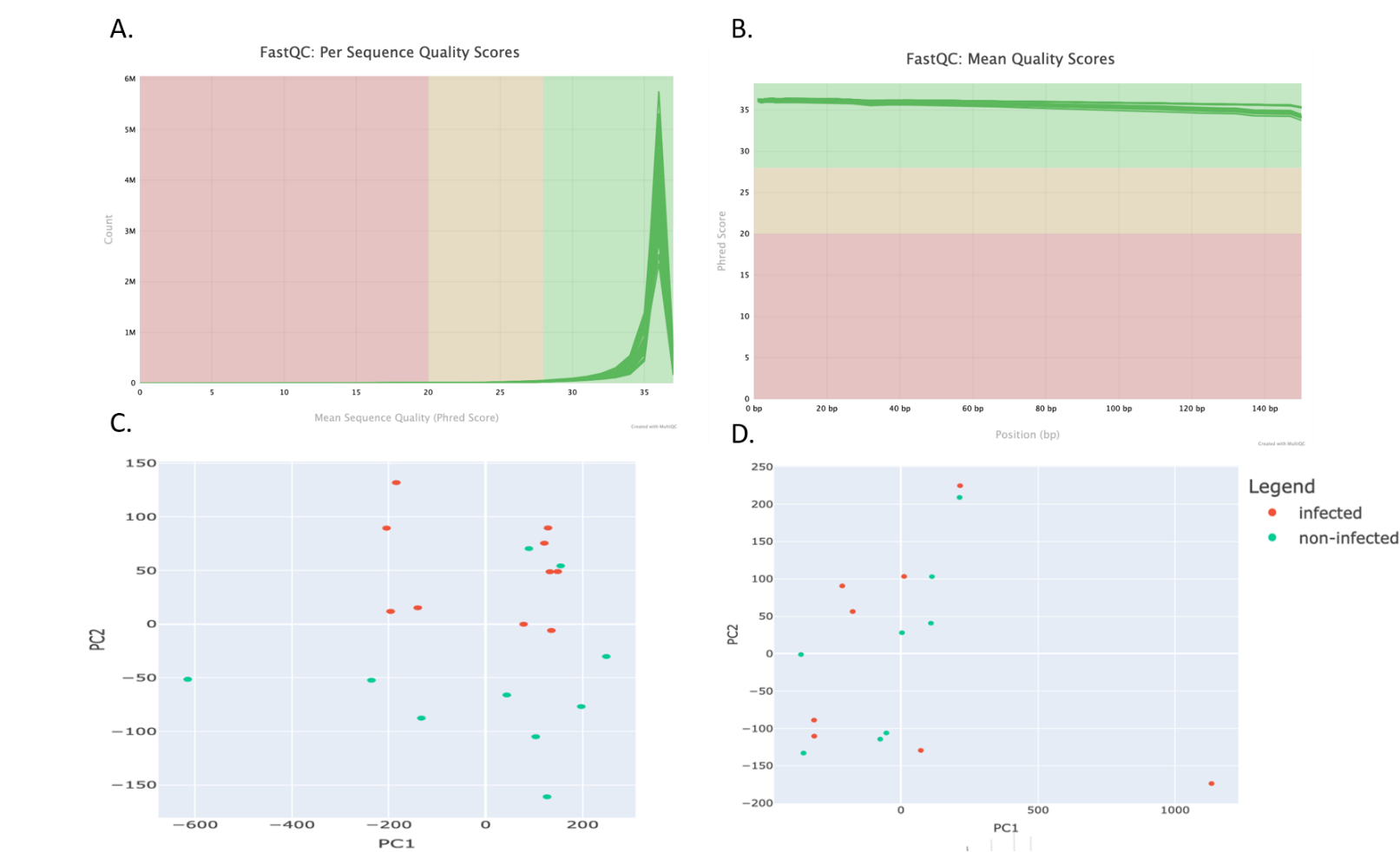


**Supplementary Figure 1. Transcriptome and proteome quality metrics and PCA.**  (**A**) Per individual RNA sequence quality scores. (**B**) Mean RNA sequence quality scores. (**C**) Transcriptome PCA. (**D**) Proteome PCA. Quality was assessed after extracting cockroach reads from raw reads and removing low-quality and unmapped reads. The cleaned cockroach reads shown in the figure had mean and individual Phred quality scores above 35.
