# Supplementary material for: Multi-omics of cockroaches infected with Salmonella Typhimurium identifies molecular signatures of vector colonization
Source: BMC Genomics. 2025 Nov 18;26:1118. doi: 10.1186/s12864-025-12333-y (PMC12752085; doi:10.1186/s12864-025-12333-y)
Supplement: Supplementary file 9 — Supplementary Material 9. Additional proteomics methods. [file 12864_2025_12333_MOESM9_ESM.docx]

**Quantitation Overview-Spectral Counting**

Below is a flow chart for how quantitation is performed for a binary comparison of two biological samples with three replicates when Total Spectral Count Normalization is applied. There are a number of normalization methods that can be applied but the default is to apply no normalization.


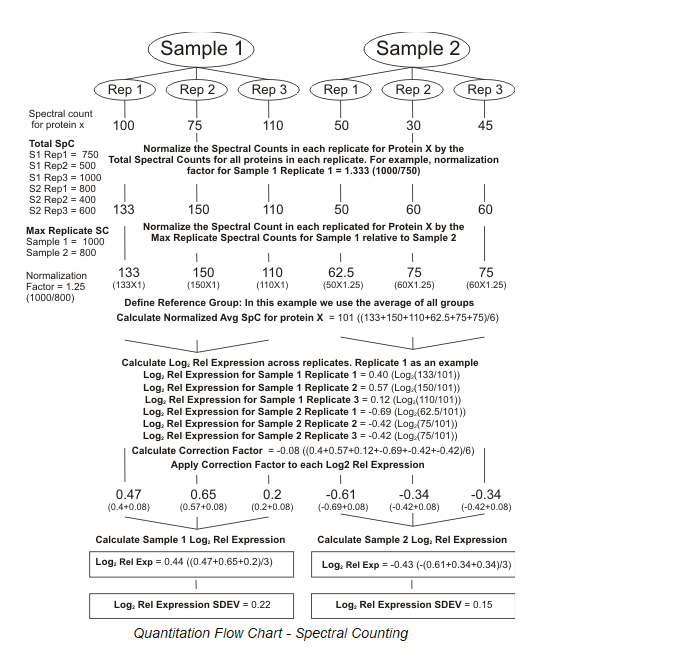


**The ProteoIQ Quantitation Workflow - Label Free**

1. Total Spectral Counts are first determined for each replicate by summing the spectral counts for all proteins identified in that replicate (shown as Total SPC)
2. The spectral counts for each protein in each replicate are then normalized based on user specifications. In this example we use Total Spectral Counts to normalize.
3. Normalization factors are calculated between Biological Samples. Designated as Normalization Factor using Max Replicate SC.
4. The Replicate Normalized spectral counts are the normalized using the Biological Sample Normalization factor. In the example above, each replicate normalized spectral count is multiplied by the Normalization Factor according to Biological Sample (either 1 or 1.25).
5. Average spectral counts are calculated for every problem across the replicates. Designated as Normalized Avg SpC. Note: By default expression ratios are calculated relative to the Normalized Avg SpC across all Biological Groups. The user has the option of changing the reference group using the Modifying Label Free Quantitation Settings.
6. Log_2_ transformations of the Relative Expression values are calculated for each protein across replicates. Relative Expressions values are calculated as the spectral count for proteins X divided by the average spectral count for that protein across all biological samples for the same replicate or using the user defined reference group.
7. Correction factors are then calculated across all groups and replicates by calculating the average Log_2_ Relative Expression across all replicates and groups. The assumption is that average Log_2_ Relative Expression across all groups should be zero. Each replicate Log_2_ Relative Expression is then corrected based on the average Log_2_  value.
8. To determine the Log_2_ Relative Expression for a Biological Sample the Replicate Log_2_ relative expression values are averaged across all replicates within a biological sample.
9. Standard deviations for the Log_2_ Relative Expression are then reported.

**Total Spectral Count Normalization**

In many cases over sampling of a proteome of a one biological sample relative to another biological sample can occur. In theses cases normalizing the spectral counts for each protein in the under sampled proteome relative to the over sampled state can make the quantitation more accurate. ProteoIQ performs this normalization by comparing the total spectral counts for all proteins identified in each biological group and replicate. The normalization factors are calculated such that the total spectral counts for all proteins in each replicate and biological sample are equal. The normalization factors are then applied to the spectral counts for each protein. See Quantitation Overview – Spectral Counting for a detailed description of how normalization is applied in ProteoIQ.

The normalization dialog can be accessed from the menu bar under Edit> Edit Normalization Settings

Note: When accessed in this manner the User – Specified Control Proteins button is not available.

Customize Normalization Options:

1. Click the box for spectral Count Normalization
2. Choose the normalization method – Total sampling in biological groups and replicates.
3. The normalization factors for each replicate and biological sample will be displayed.
4. Specify a Protein Set to use the spectral count normalization factors if applicable. If the selection is “None” then the total spectral counts from all parsed peptides will be used. Selection of a specific protein set indicates that the total spectral counts will be calculated for the specified protein set and not the whole proteome.
5. Click apply
6. To recalculate Log_2_  expression values using the selected normalization factors you MUST CREATE A NEW PROTEIN SET
7. To create a new protein set either
8. Highlight all of the proteins, right click and select copy to new protein set or
9. Select the protein set in the Filter Previous Set drop down menu and click Create New Protein Set

**Normalized Spectral Abundance Factors (NSAF)**

Protein Normalized Spectral Abundance Factors (NSAF) are calculated as the number of spectral counts for proteins x (SpC) divided by the number of amino acids (L) in protein x divided by the sum of SpC/L for all proteins in the experimental data set.


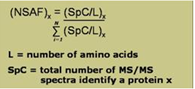


Spectral count normalization based on protein size and protein set composition has been shown to improve the accuracy of spectral count quantification (see references below) NSAF values have also been applied to study protein complexes and generate protein interaction networks.

NSAF normalizes to a relative abundance across proteins within each biological group. ProteoIQ multiplies the NSAF by the protein spectral count order to preserve the sampling rate. This maintains the sampling differences across groups so that protein expression can then be compared between them. Following any other normalization procedures the NSAF is applied, and spectral count quantitation is carried out as described in.

The normalization dialog can also be accessed from the menu bar under Edit> Edit Normalization Settings

Note: when accessed in this manner the User- Specified Control Proteins button is not available.

Customize Normalization Options:

1. Choose the normalization method _Normalized Spectral Abundance Factors
2. Specify a Protein Set to use for the spectral count normalization factors if applicable. If the selection is “None” then the total spectral counts from all parsed peptides will be used. Selection of a specific protein set indicates that the total spectral counts will be calculated for the specific protein set and not the whole proteome
3. Click Apply
4. To recalculate Log_2_ expression values using the selected normalization factors you MUST CREATE A NEW PROTEIN SET
5. To create a new protein set either
6. Highlight all of the proteins, right click and select copy to new protein set or
7. Select the protein set in the Filter Previous Set drop down menu and click Create New Protein Set

References:

- Boris L. Zyballov, Laurence Florens and Michael P. Washburn. Quantitative shotgun proteomics using a protease with broad specificity and normalized spectral abundance factors. Mol. BioSyst ,2007, 3, 354-360
- Boris Zyballov, Amber L. Mosley, Mihaela E. Sardiu, Michael K. Coleman, Laurence Florens, and Michael P. Washburn. Statistical Analysis of membrane Proteome Expression Changes in Saccharomyces cerevisiae. J. Proteome Res, 2006,05(9), pp 2339-2347
- David W. Powell, Connie M. Weaver, Jennifer L. Jennings, K. Jill McAfee, Yue He, P. Anthony Well, and Andrew J. Link. Cluster Analysis of Mass Spectrometry Data Reveals a Novel Component of SAGA. Molecular and Cellular Biolog. August 2004, p.7249-7259
